# DACH1 inhibits cyclin D1 expression, cellular proliferation and tumor growth of renal cancer cells

**DOI:** 10.1186/s13045-014-0073-5

**Published:** 2014-10-17

**Authors:** Qian Chu, Na Han, Xun Yuan, Xin Nie, Hua Wu, Yu Chen, Mingzhou Guo, Shiying Yu, Kongming Wu

**Affiliations:** Department of Oncology, Tongji Hospital, Tongji Medical College of Huazhong University of Science and Technology, 1095 Jiefang Avenue, Wuhan, Hubei 430030 China; Department of Gastroenterology & Hepatology, Chinese PLA General Hospital, #28 Fuxing Road, Beijing, 100853 China

**Keywords:** Renal carcinoma, DACH1, Tumor growth, Proliferation, Cell cycle, Cyclin D1

## Abstract

**Background:**

Renal cell carcinoma (RCC) is a complex with diverse biological characteristics and distinct molecular signature. New target therapies to molecules that drive RCC initiation and progression have achieved promising responses in some patients, but the total effective rate is still far from satisfaction. Dachshund (DACH1) network is a key signaling pathway for kidney development and has recently been identified as a tumor suppressor in several cancer types. However, its role in renal cell carcinoma has not been fully investigated.

**Methods:**

Immunohistochemical staining for DACH1, PCNA and cyclin D1 was performed on human renal tissue microaraays and correlation with clinic-pathological characteristics was analyzed. *In vitro* proliferation, apoptosis and *in vivo* tumor growth were evaluated on human renal cancer cell lines with decitabine treatment or ectopic expression of DACH1. Downstream targets and potential molecular mechanism were investigated through western blot, immunoprecipitation and reporter gene assays.

**Results:**

Expression of DACH1 was significantly decreased in human renal carcinoma tissue. DACH1 protein abundance was inversely correlated with the expression of PCNA and cyclin D1, tumor grade, and TNM stage. Restoration of DACH1 function in renal clear cell cancer cells inhibited *in vitro* cellular proliferation, S phase progression, clone formation, and *in vivo* tumor growth. In mechanism, DACH1 repressed cyclin D1 transcription through association with AP-1 protein.

**Conclusion:**

Our results indicated that DACH1 was a novel molecular marker of RCC and it attributed to the malignant behavior of renal cancer cells. Re-activation of DACH1 may represent a potential therapeutic strategy.

## Introduction

Renal cell carcinoma (RCC) is the most lethal type of genitourinary cancer and its incidence has been increased worldwidely [1]. Lacking specific markers makes early diagnosis difficult. Prognosis for advanced RCC is poor because of highly metastatic and generally resistant to conventional chemotherapy and radiotherapy [2]. With the growing understanding of renal cancer biology, new agents targeting specific growth pathways have been developed. The mammalian target of rapamycin (mTOR), a serine/threonine protein kinase, regulates cell growth, division, and survival. Clinically, mTOR inhibitors have clearly shown survival advantage than interferon-alpha [3]. Most renal clear cell carcinomas showed enhanced angiogenesis, and targeting vascular endothelial growth factor (VEGF) with either tyrosine kinas inhibitors or anti-VEGF monoclonal antibody also demonstrated superior activity in comparison to traditional chemotherapies [4]. However, even treated with the newest targeted therapeutic agents, metastatic RCC will progress in all patients due to primary or secondary resistance [5]. Obviously, RCC is a complex with diverse biological characteristics and distinct molecular signature. Many other biological factors may influence the therapeutical response of RCC. Accurate pathological characterization will guide the clinical management of RCC. Therefore, new molecular markers to stratify patient risk and predict patient response to therapy for personalized medicine can further bring survival benefits [6,7].

The characterization of renal cell carcinoma based on gene expression patterns has the potential to supply significant biological and clinical insights. Kidney cancers show aberrant methylation and methylation profiles can be predictive of adverse prognosis [8]. DNA hypermethylation in CpG islands of promoter region usually results in transcriptional silencing, a common mechanism leading to the inactivation of tumor suppressor. In the search for novel epigenetic markers for clear cell renal cell carcinoma, Dr. Dalgin’s group found DACH1 was among the 6 down-regulated genes with hypermethylation of promoter region [9].

DACH1, originally discovered in drosophila eye development, is an essential member of **R**etinal **D**etermination **G**ene **N**etwork (RDGN) [10]. RDGN mainly consists of Dach, Eya and Six family members. Balanced functions of RDGN are essential for normal development of many organs, including kidney and ear [11]. Recently, altered expressions or activity of the RDGN has been documented in a variety of malignancies [12-14]. Generally, DACH1 behaves as a tumor suppressor, and its expression is reduced in several cancers. The ectopic expression of DACH1 inhibits cellular proliferation *in vitro* and tumor growth *in vivo* [15-23]. On the other hand, Six and Eya are frequently overexpressed and promote proliferation, invasion and tumorigenesis [24-28]. It is important that expression level of DACH1 can predict survival in breast cancer [15,29]. RNA protection assay and northern blot indicated that DACH1 was richly expressed in embryonal kidney cells and adult kidney tissues, but dramatically decreased in two renal cancer cells [30]. Epigenetic silencing of DACH1 mRNA was also observed in renal cancer tissues [9]. However, there were no experimental evidence and detailed clinic studies to examine the role of DACH1 in renal cancer initiation and progression. The biological function and downstream targets of DACH1 are cell context-dependent. For example, the paracrine signal repressed by DACH1 in glioma stem cells was FGF2 [19]; while DACH1 targets IL-8 in breast cancer cells [17]. The clinical significance and downstream signaling of DACH1 in RCC remain to be experimentally answered. The current study was conducted to analyze the DACH1 expression in relation to clinic-pathological characteristics and identify molecular targets of DACH1 in renal cancers.

## Results

### Decreased expression of DACH1 correlates with tumor progression in renal cancer tissues

**Figure 1:**
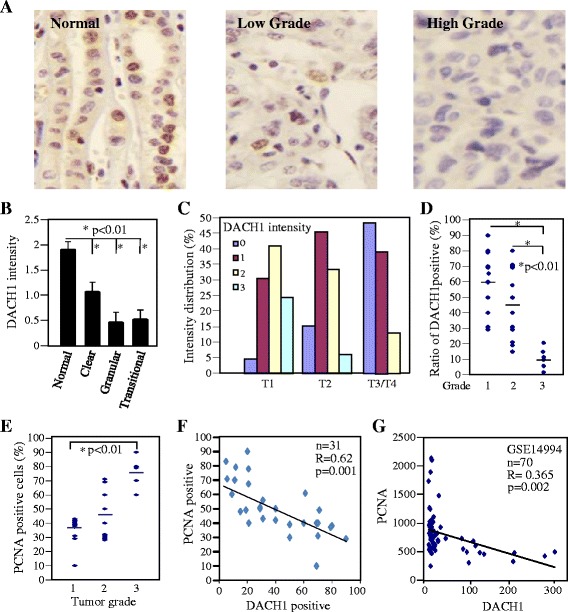
**DACH1 expression decreased in renal cell carcinoma and inversely correlated with PCNA. A**. Representative expression of DACH1 in normal kidney tissues and renal clear cell carcinomas. Intensity of DACH1 protein in relation to tumor type **(B)** and tumor stage **(C)**. **D**. The ratio of DACH1 positive cells with tumor grade. **E**. Ratio of PCNA positive cells with tumor grade. **F**. Relative protein abundance of DACH1 with PCNA. **G**. Reciprocal quantitave mRNA expression of DACH1 and PCNA in renal clear cell carcinoma tissues from Oncomine database GSE14994.

As a potential tumor suppressor, DACH1 promoted hypermethylation and correspondingly reduced expression of DACH1 was observed in several kinds of cancers, including esophageal cancer, gastric cancer, colorectal cancer and hepatocellular carcinoma [20,22,31,32]. Epigenetics changes in 38 matched renal clear cell carcinoma and normal tissues demonstrated that DACH1 promoter region was hypermethylated in renal cell carcinoma [9]. To the best of our knowledge, there were no reports that comparing DACH1 protein abundance between renal normal and cancerous tissues. We used a well validated DACH1 polyclonal antibody to detect DACH1 expression in human renal tissue microarrays consisting of normal and different types of cancers by immunohistochemical staining. DACH1 was highly expressed in the nuclei of renal tubular cells. Although RCC originates from the tubule of kidney, DACH1 expression was markedly decreased in all 3 major types of renal cancers, including clear cell renal carcinoma and granular cell carcinoma (Figure 1A, B). Further analysis showed that DACH protein intensity was gradually reduced with the tumor progression. More than 85% tissues in T3/T4 tumors showed no or very weak expression(grade 0 or 1); while in early-stage tumors (T1), 65% tissues had medium or strong expression (grade 2 or 3) (Figure 1C). Moreover, on average 60% of cells in low grade cancers (grade I) expressed DACH1, less than 20% cells in grade III tumors had detectable DACH1 expression (Figure 1D). Thus the DACH1 expression was significantly reduced in cancer tissues, correlated inversely with the tumor grade and stage. Since the high proliferation is a hallmarker of cancer cells and DACH1 was reported to inhibit tumor growth *in vivo* in a series of xenograft models [15,19,23], we examined PCNA expression, a surrogate marker of cellular proliferation, in a series of sections from the same sample. In consistence with previous reports, PCNA was positively related to the tumor grade (Figure 1E). Importantly, co-expression analysis demonstrated reverse relationship between protein expression of DACH1 and PCNA in renal cancer tissues (Figure 1F). In order to further investigate the relationship of DACH1 and PCNA at mRNA level, we examined Oncomine database. The mRNA profiles GSE14994 consisting of 70 patients with renal cancers showed that DACH1 and PCNA were inversely correlated (Figure 1G) (p < 0.002). Therefore, we conclude that the lost expression of DACH1 led to higher cellular proliferation in renal cancer tissues.

### Reactivation of DACH1 expression by methylation inhibitor reduced renal cancer cellular proliferation

**Figure 2:**
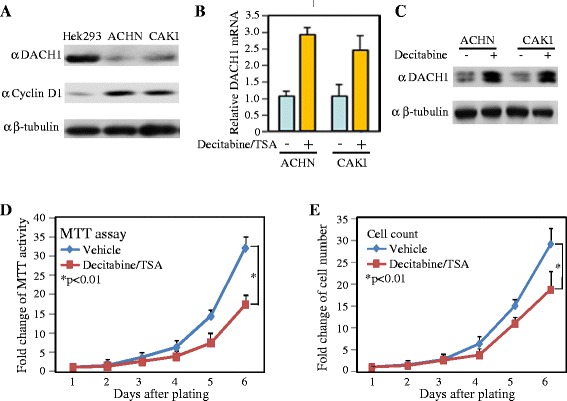
**Epigenetic silencing of DACH1 enhanced cellular proliferation. A**. Western blot analysis of DACH1 and cyclin D in embryo kidney cells and clear cancer cells. **B**. Relative DACH1 mRNA expression normalized to GAPDH with or without treatment. **C**. Protein abundance with or without treatment. β-tubulin as loading control. Cellular proliferation analyzed by MTT assay **(D)** and cell counting **(E)**.

DACH1 mRNA was highly expressed in several adult tissues including kidney, heart, lung and brain, with the highest expression detected in adult kidney tissues [30]. Using the embryo kidney cell (HEK293) as positive control, we determined DACH1 abundance in two clear cell cancer lines ACHN and CAKI. Western blot showed that DACH1 was very weakly expressed in both cancer cell lines, in contrast DACH1 was abundantly expressed in HEK293 cells (Figure 2A). After sequentially treated with Decitabine in combination with Trichostatin A (TSA), DACH1 mRNA was induced about 3 folds increase (Figure 2B). Correspondingly, DACH1 protein was increased about 5 folds (Figure 2C). Cellular proliferation ability was evaluated in ACHN cells treated with Decitabine and TSA. Both MTT assay and cell counting demonstrated that combined treatment reduced the cancer growth rate (Figure 2D, E). Those results indicated that epigenetic silencing of endogenous DACH1 contributed to the enhanced growth of RCC cells.

### Ectopic expression of DACH1 inhibited renal cancer cell in vitro proliferation and in vivo tumor growth

**Figure 3:**
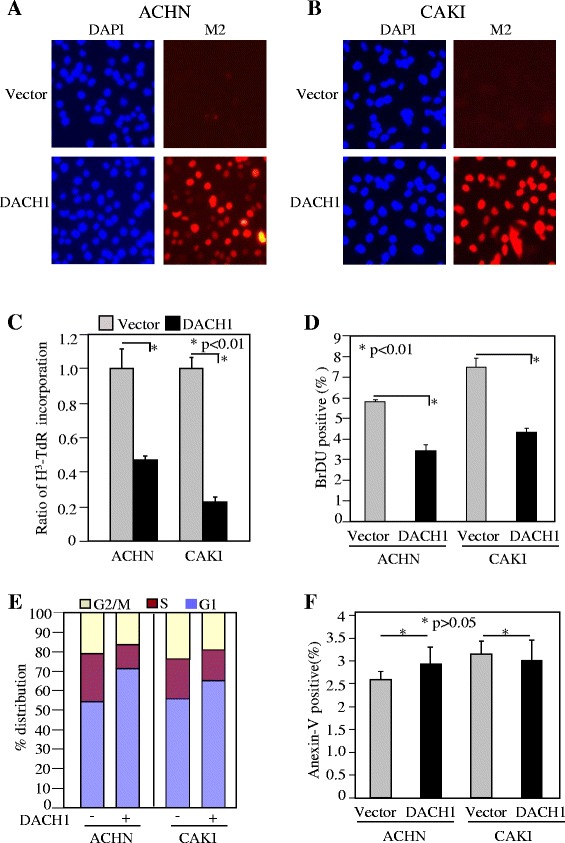
**Ectopic expression of DACH1 inhibited cellular proliferation**. Stable expression of Flag-taged DACH1 in renal cancer cell lines ACHN **(A)** and CAKI **(B)**. DNA synthesis evaluated by H^3^-TdR incorporation **(C)** and BrdU incorporation **(D)**. Proliferation evaluated by S phase entry **(E)** and apoptosis evaluated by Annexin-V stanining **(F)**.

**Figure 4:**
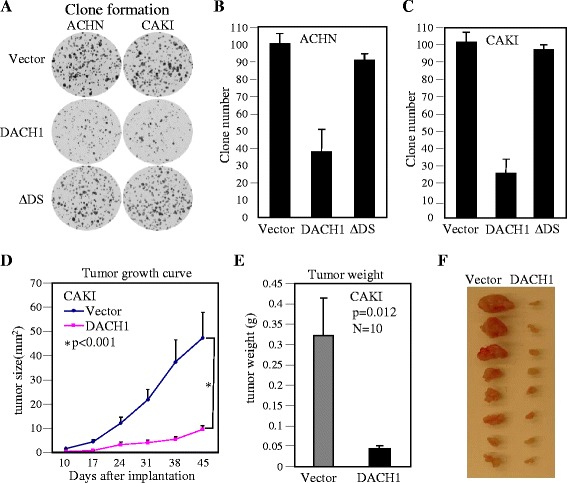
**DACH1 inhibited clone formation and tumor growth** ***in vivo*** **. A**. Representative images of clone formation. Relative clone formation efficiency of 3 experiments for ACHN **(B)** and CAKI **(C)**. **D**. Tumor growth curve of subcutaneously implanted CAKI cells. **E**. The tumor weight after 45 days. **F**. Images of tumors from CAKI cells with or without DACH1 expression.

In order to directly define the function of DACH1, we established sublines through infecting ACHN and CAKI cells with retrovirus expressing DACH1. Two weeks after antibiotic selection, more than 90% cells expressed Flag-tag DACH1 as shown by fluorescent staining (Figure 3A, B). Expression of DACH1 decreased the cell proliferation in both ACHN and CAKI cells (Figure 3C, D). Flow cytometry revealed that the decrease in S phase was corresponded to the increase in G1 (Figure 3E). However, there was no statistical difference in apoptotic cells with or without DACH1 expression (Figure 3F). The clone formation is a basic characteristic of transformed cells and represents the malignant potential and tumorigenicity. Engineering expression of wild type DACH1 inhibited the clone number in both ACHN and CAKI cells, in contrast, expressing a DACH1 with DS domain deleted mutant had no repressive function (Figure 4A, B, C). In order to further evaluate tumorigenecity *in vivo*, CAKI cells expressing DACH1 and the vector control were subcutaneously implanted to immunodeficient mice and the tumor growth was monitored twice a week. The growth curve revealed a dramatic decrease of tumor size in the CAKI group following DACH1 expression (Figure 4D). The average tumor weight decreased from 320 mg in the control group to 50 mg in the DACH1 group (Figure 4E, F). Moreover, gross observation indicated that cancer cells in vector group infiltrated into surrounding host tissues, while tumors in DACH1 group was not only smaller, but also clearly separated from surrounding host tissues.

### DACH1 repressed cyclin D1 in vitro and in vivo

**Figure 5:**
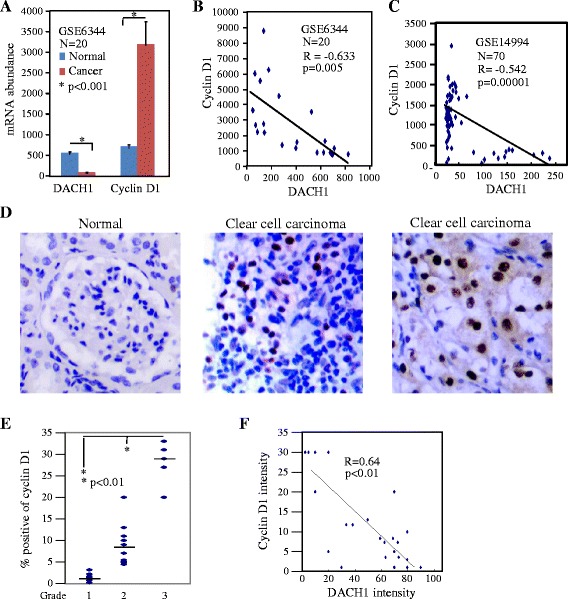
**DACH1 inversely correlated with cyclin D1 in renal cancer tissues. A**. Quantitative mRNA expression of DACH1 and cyclin D1 in normal renal tissues and renal carcinomas. Reciprocal expression of DACH1 and cyclin D1 from Oncomine database GSE6344 **(B)** and GSE14994 **(C)**. **D**. Representative image of cyclin D1 in normal renal tissues and clear cell carcinoma tissues. **E**. Cyclin D1 expression with tumor grade. **F**. Reciprocal protein abundance of DACH1 and cyclin D1.

Previous studies proved that RDGN integrated with cell cycle regulatory machinery to modulate cellular proliferation and tumorigenecity in several types of cancers [15,24,33-35]. Specifically, Six1 and EYA1 upregulated cyclin D; In contrast, DACH1 acted as an antagonist of cyclin D1 in breast cancer. We searched in Oncomine database to find whether this reciprocal relation exists in renal cancer. Quantitative mRNA expression from 20 pairs of normal and cancerous tissues showed that higher DACH1 expression is accompanied with lower cyclin D1 expression in normal renal tissue. However, DACH1 was reduced more than 80% in cancerous tissues with up to 6 folds increase in cyclin D (Figure 5A). The reverse correlation was observed in each sample from two different databases (Figure 5B, C). To further explore their relationship in the protein level, human renal cancer tissue microarrays were immunostained with antibodies for DACH1 and cyclin D1. Very few cells were positive for cyclin D1 in normal renal tissue, and cancer cells expressed cyclin D1 at different ratio (Figure 5D). The ratio of cyclin D1 positive cells statistically increased with the tumor grade (Figure 5E). Moreover, the higher expression of DACH1 usually accompanied with the lower expression of cyclin D1, with coefficient R value of −0.64 (p < 0.01) (Figure 5F).

**Figure 6:**
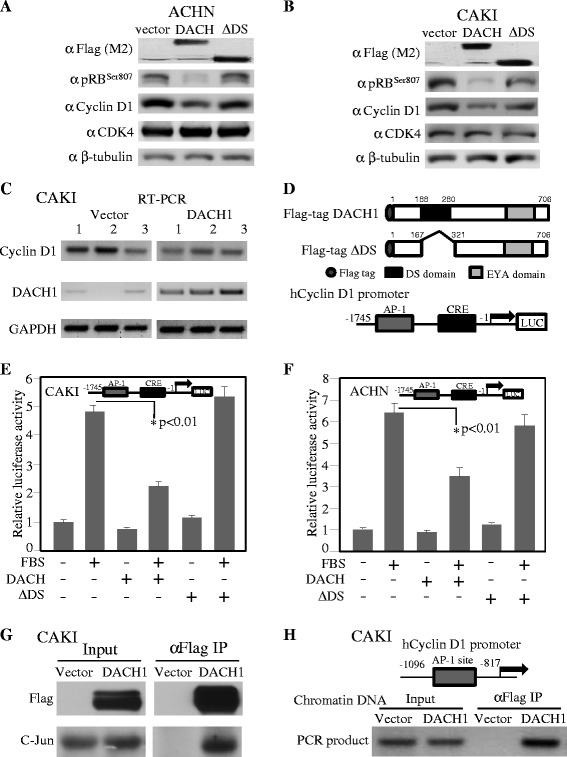
**DACH1 in association with c-Jun to repress cyclin D1 transcription.** Western blot analysis of ACHN **(A)** and CAKI **(B)** cells expressing DACH1 wt, DS or vector control. **C**. RT-PCR detected cylin D1 and DACH1 mRNA abundance. **D**. Schematic structure of DACH1 and cylin D1 promoter. **E**. Luciferase activity of cyclin D1 promoter in renal cancer cell line CAKI **(E)** and ACHN **(F)**. **G**. Immunoprecipitation and western blot of CAKI cells. **H**. ChIP of cyclin D1 promoter AP-1 site in CAKI cells.

The cyclin D1 and related cell cycle protein RB and CDK4 were measured in cultured renal cancer cell lines. Western blot results demonstrated that ectopic expression of wild type DACH1 dramatically repressed cyclin D1 and phosphorylated RB proteins, but it had no effect on CDK4 in both ACHN and CAKI cells (Figure 6A, B). RT-PCR demonstrated about 50% decrease of cyclin D1 mRNA in CAKI cells expressing DACH1 (Figure 6C). To measure the transcriptional regulation of cyclin D1, transient co-transfection assay was performed in ACHN and CAKI cells using DACH1 expressing vector and cyclin D1 promoter constructs linked with luciferase (Figure 6D). As a positive control, serum activated cyclin D1 promoter activity was increased about 5–6 folds; while DACH1 reduced cyclin D1 promoter activity to less than 50%. However, a DS domain with deleted mutants abrogated repressive function (Figure 6E, F). Immunoprecipitation with anti-flag antibody and western blot using a c-Jun antibody in the whole cell lysate demonstrated DACH1 formed a complex with c-Jun in CAKI cells (Figure 6G). Chromatin immunoprecipitation with a flag antibody and PCR amplified human cyclin D1 promoter sequence supported a model that DACH1 was recruited into cyclin D1 promoter context at AP-1 site, as previously observed in human breast cancer (Figure 6H) [15].

## Discussion

The current studies intended to demonstrate DACH1 protein expression was significantly reduced in RCC tissues in comparison to normal kidney tissues. Moreover, the DACH1 protein level inversely correlated with tumor grade and TNM stage. This was in agreement with previous findings that DACH1 mRNA in RCC tissues was 80% less than the matched normal kidney tissues due to the hypermethylation of DACH1 promoter region [9]. This finding was also in accordance with most observations in solid tumors and further supported the concept that DACH1 represented a novel tumor suppressor [15,16,19-21]. Functional inactivation of tumor suppressors by promoter methylation is a common mechanism of tumorigenesis. Histone deacetylase inhibitors (HDACI) had great anti-cancer effect in a wide range of cancers in preclinic studies and exhibited promising responses in patients with various cancer types [36]. In this respect, CDF, a new synthetic analogue of curcumin, had been demonstrated as a novel demethylating agent for restoring the expresson of hyper-methylated gene and caused a marked inhibition of cellular growth [37]. We showed that decitabine treatment induced the expression of DACH1 and inhibited cellular proliferation. However, whether DACH1 is the key target of decitabine for *in vivo* tumor growth needs to be further evaluated. In contrary to epigenetic changes and mRNA expression, previous studies failed to reveal the decreased protein abundance of DACH1 in cancer tissues [9]. DACH1 has 3 isoforms and its isoform expressions showed a tissue-specific pattern [23]. Both antibody specification and isoform expression may account to this discrepancy.

Previously, we showed that DACH1 was lost in triple negative breast cancers associated with stem cell property, and high expression of DACH1 predicted a 40-month survival advantage in all types of breast cancer [15]. Recently, Dr. Powe’s study demonstrated that high expression of DACH predicted a better survival in luminal breast cancers [29], further supporting our previous report. However, the prognostic value of DACH1 in RCC remains to be elucidated.

Our studies demonstrated that the expression of DACH1 inversely correlated with cyclin D1 and PCNA, the surrogated markers of proliferation. It suggested that the loss of DACH1 led to a growth advantage of tumor cells. The sequential epigenetic treatment with epigenetic modification agents decitabine/TSA induced DACH1 expression accompanied with decreased proliferation, providing a laboratory evidence to support the concept that inactivation of DACH1 contributed to tumor growth. Indeed, the engineering expression of DACH1 inhibited cell growth via the reduced DNA synthesis, not the altered apoptosis, depending on the DS domain. Our results were in agreement with previous findings in other cell lines that the inhibition of cellular proliferation by DACH1 required the key DS domain. The DS domain has been proved to be required for a series of important functions, including the regulation of cancer stem cell expansion [38], AR [16], IL-8 secretion and breast cancer cell invasion [17]. Transcriptional repression of AP-1 family members c-jun and c-fos by DACH1 also required the conserved N-terminal DS domain. The deletion of DS domain abolished the repression of Cyclin D1 in breast cancer and the overexpression of DS domain alone substituted DACH1 to repress S phase entry in breast cancer cells [15]. At the molecular level, the DS damin alone can be recruited to the cyclin D1 promoter AP site. In association with AP-1 and smads complex, DS domain possessed DNA binding property [18]. DACH1 antagonized FOXM1 signaling through competitively binding to the conserved forehead specific DNA sequence [18]. Together, those experiments demonstrated that DACH1 DS domain and its associated proteins play key role in the DACH1-mediated function. Molecules or reagents targeting to this portion of DACH1 may have potential therapeutical applications.

The *cyclin D1* gene encoded the regulatory subunit of a holoenzyme that phosphorylated and inactivated the pRb protein, thereby promoting the DNA synthesis and the S phase entry. Aberrations in the G1/S transition of cell cycle were observed in many cancers. pVHL downregulated cyclinD1 through HIF-independent mechanisms [39], therefore, conventional RCC often expressed high cyclin D1 protein level [40]. Cyclin D1 was a key downstreaming target of mTOR. In RCC cell line CAKI and ACHN, dual inhibition of src kinase and receptor tyrosine kinase resulted in synergistic inhibition of proliferation and migration, accompanied with suppression of cyclin D1 [41]. Moreover, tyrosine kinase inhibitor, sorafenib, inhibited angiogenic downstream signaling p-AKT, p-ERK and cyclin D1 [42]. Metoformin inhibited the proliferation and tumor growth of RCC cell lines 786-O and OS-RC-2, also with down-regulating of cyclin D1 expression and cell cycle arrest [43]. Experimentally, exposure of human renal cells to recombinant erythropoietin induced cellular proliferation through stimulating the expression of cyclin D1 while inhibiting the expression of p21^cip1^ and p27^kip1^ [44]. Approximately 75% of the RCC tumors expressed higher level of cyclin D1 than the normal kidney context. Surprisingly, previous studies did not find cyclin D1 expression correlated with proliferation, as determined by Ki-67 or s-phase analysis [45].

The abundance of cyclin D1 was regulated through distinct mechanisms, including post translational modification by phosphorylation and the induction of mRNA and/or gene transcription. Our data indicated DACH1 repressed cyclin D1 transcription, as determined by RT-PCR and promoter activity assays. Recent studies revealed multiple novel functions of cyclin D1, including cancer stem cell self-renewal [46], VEGF-stimulated vascularization [47] and chromosomal instability [48]. Considering cyclin D1 as the common downstream target of multiple signaling in RCC target therapy, the current studies raised the possibility that expression level of DACH1 may determine targeting therapeutic sensitivity either directly or indirectly, therefore, affect the long term survival.

p53 is a powerful tumor suppressor and small molecules that activated p53 had shown promising anti-tumor effects in hematological malignancies [49]. Recent findings that DACH1 associated with and enhanced p53 function to induced apoptosis provided an alternative mechanism for DACH1 to inhibit tumorigenesis [21]. However, as a new tumor suppressor, detailed targets of DACH1 and its crosslinks with other oncogene/tumor suppressors remain to be further clarified.

## Materials and methods

The study protocol was approved by the ethics committee of Tongji Medical College of Huazhong University of Science and Technology.

### Cell culture

Human renal clear cell carcinoma cell lines CAKI-1 were cultured in McCoy’s 5A medium with 10% fetal bovine serum. ACHN cells were cultured in EMEM supplemented with 2 mM L-glutamine, 1% non-essential amino acids, 1 mM sodium pyruvate, and 10% fetal bovine serum. Human embryonic kidney 293 cells (HEK293) were maintained in DMEM containing 1% penicillin/streptomycin and supplemented with 10% FBS.

### Plasmid construction, stable cells, reporter genes, DNA transfection and luciferase assay

Expression plasmids for DACH1 and DACH1 DS domain deleted mutation (ΔDS) in pKW10 vector were a gift from Dr. Cvekl [50]. After digestion with ClaI/EcoRV, the insert was subcloned into a retrovirus expression vector. Stable sublines expressing DACH1, ΔDS and empty vector control were established by transient co-transfection of DACH1-expressing vector with package plasmids in HEK293T cells. At 36 h and 48 h after transfection, the culture medium was collected and filtered through a 0.45 μm filter for infecting renal cancer cells in the presence of 8 mg/ml polybrene. The pool of transduced cancer cells was further selected by treatment with Blastcidin at 5ug/ml for 2 weeks. Human cyclin D1 promoter constructs were a gift from Dr. Pestell [15]. Plasmid DNA transfection and luciferase assays were performed as previously described [15].

### RNA isolation, RT-PCR and quantitative real-time PCR

Total RNA was isolated from CAKI cells stably expressing DACH1 and vector control using the TRIzol reagent (Invitrogen) following the manufacturer’s instructions. Five micrograms of total RNA was subjected to DNase treatment and purification following RNA minipre kit (Qiagen). cDNA was synthesized using the SuperScript II Reverse Transcriptase Kit (Invitrogen). RT-PCR primers for cyclin D1 were: 5’-GTGCTGCGAAGTGGAAACC -3’ and 5’- ATCCAGGTGGCGACGATCT-3’ [33]; primers for DACH1 were: 5’-CCA TGA GCA ACT ATC ATG CC-3’and 5’-TGT CCA TGC CCA GTT AGA GA-3’ [16]. Internal control primers for GAPDH were 5’- ATCTTCCAGGAGCGAGACCCC-3’ and 5’- TCCACAATGCCAAAGTTGTCATGG-3’.

### Immunohistochemistry and immunofluorescence

Human kidney cancer tissue arrays were purchased from Alenabio (Xi’an, China), including 13 cases of normal renal tissues, 5 cases of cancer adjacent normal renal tissue, 55 cases of clear cell carcinoma tissue, 31 cases of granular cell carcinoma tissue, and 19 cases of transitional cell carcinoma tissue. Tumor tissues were sub-grouped as grade I, well-differentiated; grade II, moderately differentiated; and grade III, poorly or undifferentiated. TNM status was as follows: 66 cases of T1N0M0, 33 cases of T2N0M0, 9 cases of T3N0M0 and 2 cases of T4N0M0. Tissue immunohistochemical stains were performed by core facility using a streptavidin-biotin technique and semi-quantated as described [51]. The antibodies were polyclonal antibody to DACH1(Proteintech, 10914–1), to PCNA (Santa Cruz, sc-7907) and monoclonal antibody to Cyclin D1 (Santa Cruz, sc-20044). The immune stain intensity and ratio of positive cells were analyzed. Immunofluorescence staining for DACH1 was modified from published method [52]. Cells were fixed in 4% formaldehyde for 10 minutes, permeable by 1% Triton X-100 for 5 minutes then blocked in 5% goat serum for 1 hour. Primary anti-Flag antibody (M2, Sigma, F3165) was used at 1:200 dilution, the goat anti-mouse secondary antibody (Alexa Fluor-568) was used at 1:500. Cell nuclei were count-stained with 4,6-diamidino-2-phenylindole(DAPI).

### Western blot, immunoprecipitation and chromatin-immunoprecipitation study

Cultured cells at 90% confluence were pelleted and lysed in RIPA Buffer (150 M NaCl, 20 mM Tris-HCI, 1 mM EDTA, 1 mM EGTA, 1 mM Na_3_VO_3_, 2.5 mM sodium pyrophosphate, 1 mM β-glycerophosphate, 1% Triton x-100), supplemented with protease inhibitors. Protein was separated by a 10% SDS PAGE and antibodies used in Western blot included cyclin D1 (sc-20044), CDK4 (sc-601), c-Jun (sc-1694), pRB^ser807^ (Sigma, R3903), and β-actin (sc-47778) as an internal loading control. Immunoprecipitation for protein complex of DACH1 and c-Jun and in renal cancer cells was performed as previously described [15]. Chromatin immunoprecipitation(ChIP) was performed using published method [24]. The human cyclin D1 promoter-specific primers used were as follows: AP-1 site:5’-GGCAGAGGGGACTAA TATTTCCAGCA-3’ and 5’-GAATGGAAAGCTGAGAAACAGTGATCTCC-3’. Immunoprecipitation with IgG was used as negative control.

### Cell proliferation and apoptosis assay

Cells expressing DACH1, ΔDS and vector control were seeded into 96-well plates in normal growth medium, and cell growth was measured by daily3-(4,5-dimethylthiazol-2-yl)-2,5-diphenyltetrazolium (MTT) assay as previously described [53]. For measuring the growth curve, cells were seeded into 12-well plates and serially counted for 6 to 7 days. DNA synthesis was analyzed by ^3^H-TdR incorporation. Briefly, 1 × 10^5^ cells were plated into 24-well plate and cultured for 36 hours. ^3^H-TdR (1 μCi/well) was added to each well and the culture was continued for another 2 hours. Cells were washed twice with cold PBS and proteins were precipitated by incubation with 10% trichloroacetic acid for 30 minutes at room temperature (100 μL/well). After additional washes, cells were treated with 0.2 N NaOH and collected in scintillation vials. For 5-Bromo-29-Deoxyuridine (BrdU) staining, cells were labeled with 100 μM BrdU for 1 hour in regular culture medium, washed 3 times with PBS, fixed in 3.7% formaldehyde/PBS for 10 minutes, treated with 4 N HCl/1% Triton-X100 for 10 minutes, and finally washed 3 times with 0.1% NP-40/PBS. The cell suspension was incubated with mouse anti-BrdU (Sigma, B8434) at 1:1000 for 2 hours at room temperature and stained with goat anti-mouse antibody (AlexaFluor 568) at 1:1000 after washing. 10,000 cells were analyzed using a flow cytometer (BD Biosciences). Apoptotic cells were measured using a BD Pharmingen AnnexinV-PE Apoptosis Kit following the standard protocol.

### Cell cycle

Cultured cells were processed by standard methods using propidium iodide staining of cellular DNA. Samples were conducted on a FACScan flow cytometer (BD Biosciences) equipped with a 488-nm laser. Histograms were analyzed for cell cycle compartments using ModFit version 2.0 (Verity Software House, Topsham, ME). A minimum of 20,000 events for each sample were collected for statistical analysis.

### Colony formation assay

For contact-dependent growth, 4 × 10^3^ renal cancer cells were plated in triplicate into 6 cm dish and medium was changed every 3 days. The colonies after 2-week growth were visualized by staining with 0.04% crystal violet in methanol for 1 hour [16].

### Tumor implantation study

A total of 2 × 10^5^ CAKI cells expressing either vector control or DACH1 were implanted subcutaneously to 6-week old ethymic male nude mice. The tumor growth was measured weekly for 4 to 5 weeks using a digital caliper. Tumor mass was weighted after mice were sacrificed. The study protocol was approved by the ethics committee of Tongji Medical College of Huazhong University of Science and Technology.

### Statistical analysis

All data were expressed as the mean ± standard error. Statistical analysis between groups was calculated by student’s t-test. P value <0.05 was considered statistically significant.

## Conclusions

Expression of DACH1 was significantly decreased in human renal carcinoma tissue and was inversely correlated with proliferation, tumor grade, and TNM stage. Restoration of DACH1 function in renal clear cell cancer cells inhibited *in vitro* cellular proliferation and *in vivo* tumor growth. The repression of cyclin D1 transcription was a key target of DACH1 in regulating cancer cell proliferation. Our results indicated that DACH1 attributed to the malignant behavior of renal cancer cells. Re-activation of DACH1 may represent a potential therapeutic strategy.
